# Effect of Organic Modifier and Clay Content on Non-Isothermal Cold Crystallization and Melting Behavior of Polylactide/Organovermiculite Nanocomposites

**DOI:** 10.3390/polym12020364

**Published:** 2020-02-07

**Authors:** M. Jesús Fernández, M. Dolores Fernández

**Affiliations:** Department of Polymer Science and Technology, Faculty of Chemistry, University of the Basque Country UPV/EHU, Paseo Manuel Lardizábal 3, 20018 San Sebastián, Spain; mjesus.fernandez@ehu.es

**Keywords:** organomodified vermiculite, polylactic acid, nanocomposites, thermal properties, non-isothermal crystallization, kinetics, differential scanning calorimetry (DSC)

## Abstract

In clay/polymer nanocomposites, the crystallization behavior and kinetics of the polymer can be affected by the presence of clay, its content and the degree of miscibility between the clay and the polymer matrix. The effect of two different organomodified vermiculites on the non-isothermal cold crystallization and melting behavior of polylactide (PLA) was studied by differential scanning calorimetry (DSC). In the presence of vermiculites, the cold crystallization of PLA occurred earlier, particularly for the highest content of the most miscible organovermiculite with PLA. The cold crystallinity of PLA decreased at low heating rates, notably at high organoclay loadings, and increased at high heating rates, especially at low vermiculite contents. According to the crystallization half-time, crystallization rate coefficient (CRC), and crystallization rate parameter (CRP) approaches, the cold crystallization rate of PLA increased by incorporating vermiculites, with the effect being most noteworthy for the vermiculite showing better compatibility. The Mo model was successful in describing the non-isothermal cold crystallization kinetics of the PLA/vermiculite composites. The melting behavior was affected by the heating rate and the type and content of clay. The nucleating effect of the most compatible clay resulted in the less perfect crystallites. The activation energy was evaluated using the Kissinger and Takhor methods.

## 1. Introduction

Polylactide (PLA) is a linear aliphatic polyester with excellent mechanical properties, thermal plasticity and biocompatibility, that has received a lot of attention in the last decades due to its renewable origin and biodegradability [1,2,3,4,5,6,7]. However, the applications of PLA are somewhat limited by some of its properties, such as brittleness and slow crystallization [6,8]. One approach to improve some of the physical and mechanical properties of PLA is the preparation of nanocomposites in combination with nanofillers [9,10]. Pristine clays and organically modified clays have been used for this purpose [11,12,13,14,15,16]. PLA is a semicrystalline polymer, and its physical/mechanical properties are determined by the extent of crystallization and the crystalline morphology. The addition of nanofillers to a polymer matrix affects not only its mechanical properties but also the crystallization and melt behavior, and thus its applications [17,18,19,20]. Therefore, the understanding of the crystallization of both polymer and polymer composites is necessary for establishing the relationship among the processing conditions, the developed structure, and the properties of the final products.

A semicrystalline polymer is able to crystallize during cooling from the melt, called “melt crystallization”, and when heated after being quenched below the glass transition temperature, known as “cold crystallization”. Examinations concerned with the melt and/or cold crystallization behavior of PLA [21,22,23], PLA copolymers [24], PLA stereocomplex [25], and PLA/other polymer blends [26] have been investigated extensively. The crystallization behavior of PLA nanocomposites has also been studied to explore the effect of different types of inorganic fillers [27,28,29,30,31]. The effect of incorporation of nanoclays on isothermal and non-isothermal melt and cold crystallization behavior of PLA has also been studied [14,15,16,32,33,34,35,36,37,38,39]. Most of the published studies have been carried out on nanocomposites containing different organically modified montmorillonites (OMMT). In some of them, only the effect of the addition of a given OMMT on the crystallization behavior of the PLA matrix was investigated [32]. In some others, either only the effect of the OMMT loading [33,38,39] or only the effect of the degree of dispersion of the clay [16,35] were investigated. Finally, in others the effect of the OMMT content and the clay dispersion level was investigated either with a given OMMT or with two OMMTs containing different organic modifiers [14,15,34]. Nam et al. [32] prepared and studied the crystallization behavior of intercalated PLA/C_18_MMT nanocomposites. The results showed that the overall crystallization rate of neat PLA increased but the ordering of spherulites decreased after the incorporation of clay. Wu et al. [33] studied the non-isothermal cold crystallization behavior of intercalated PLA/MMT modified with methyl tallow bis(2-hydroxyethyl) ammonium nanocomposites. They reported that both the clay loadings and annealing conditions influenced the cold crystallization of PLA. The finite nucleation effect of clay promoted the crystallization, while at high clay loadings the impeding effect of clay retarded it. At the lower heating rate, crystallinity decreased in the presence of clay, while at the higher heating rate (especially in higher clay loadings) it increased, in comparison to that of neat PLA. Picard et al. [38] prepared intercalated PLA/OMMT nanocomposites and studied the non-isothermal and isothermal crystallization. The results confirmed the nucleation effect induced by the nanoclay, the enhancement of PLA crystallization (especially for high heating rates), and the formation of less perfect crystals. Di et al. [39] observed that small amounts of Cloisite 30B exfoliated in PLA matrix resulted in an increase of the crystallization rate as compared with the pure PLA, while at high organoclay content the crystallization was retarded. Pluta [16] studied the effect of OMMT (Cloisite 30B) dispersion (i.e., the nanostructure) on the crystallization phenomena of PLA upon heating from the glassy, amorphous state by DSC. The results showed that the nucleating effect of nanoclay particles on the crystallization behavior of PLA was dependent on the size of inclusions, decreasing when dispersion of the filler increased. Fukushima et al. [35] studied the effect of two different clays (Cloisite 30B and organically modified fluoro-hectorite) on crystallization behavior of PLA. The results revealed a better level of clay dispersion and a more efficient nucleation in the presence of hectorita, enhancement in the crystallization rate and extent of crystallization, especially at high clay loading, and the formation of less perfect crystals. Krikorian and Pochan [14,15] reported that the addition of a highly miscible Cloisite 30B into PLLA resulted in a lower degree of crystallinity, lower bulk crystallization rates, and much higher radial spherulitic growth rate compared to PLLA matrix and the PLLA reinforced by less miscible Cloisite 15A. Ublekov et al. [34] studied the effect of Cloisite 30B content on melting behavior and crystal structure of non-isothermal crystallized PLLA/MMT nanocomposites. The results showed that the intercalation of the clay with polymer matrix led to a higher degree of crystallinity, while in the exfoliated nanocomposite it decreased. The results of the abovementioned studies are quite disparate, showing that crystallization behavior depends on many factors. These factors include the clay loading and the dispersion morphology of the clay in the matrix.

In the present study, we used a different clay mineral from MMT—vermiculite (VMT)—and investigated the effect of the organic modifier of VMT on the melting behavior, the non-isothermal cold crystallization behavior, and kinetics of PLA matrix upon nanocomposite formation. A series of PLA/organo-VMT nanocomposites containing two different types of organic modifiers and clay content were prepared in order to evaluate the effect of the type of VMT with different degrees of miscibility with the matrix and the clay loading on the cold crystallization and melting behavior of PLA. The parameters of crystallization kinetics and the activation energy of the cold crystallization process of PLA were evaluated. 

## 2. Materials and Methods

### 2.1. Materials

**Figure 1:**
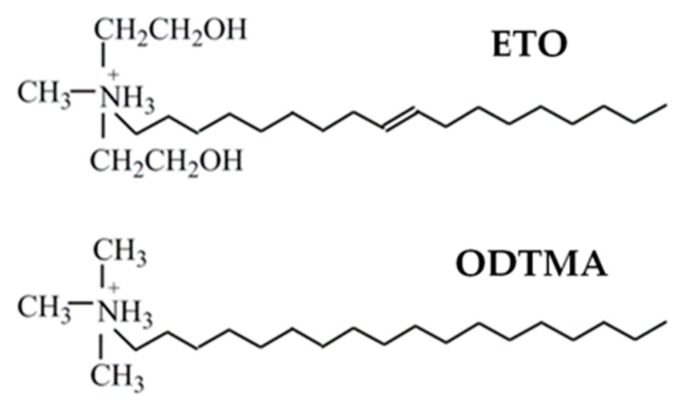
Chemical structures of organic modifiers of vermiculite (VMT).

Polylactide (PLA) (3051D) with a D-isomer content of 3.7%–4.6%, a residual monomer content less than 0.3 wt %, a number-average molecular weight of 70,000 g/mol and a polydispersity index of 1.67, as measured by gel permeation chromatography (GPC), was supplied by Nature Works LLC (Minneapolis, MN, USA). PLA pellets were vacuum dried for 2 days at 60 °C and kept in desiccation before use. Expanded vermiculite (VMT) was purchased from Sigma-Aldrich (Munich, Germany) with grade number 3, and was ground to obtain particles less than 40 μm in size. The surfactants used, oleyl bis(2-hydroxyethyl) methyl ammonium chloride (ETO) (Figure 1) supplied by Akzo Nobel (Stenungsund, Sweden), and octadecyl trimethylammonium bromide (ODTMA) (Figure 1) supplied by Aldrich (Munich, Germany), were used as received.

### 2.2. Preparation of Organovermiculites

The organo-VMTs were prepared by a cation-exchange reaction with the two different quaternary ammonium salts as described elsewhere [40,41]. The required amount of ETO, 1.25 equivalent of the CEC, dissolved in deionized water at 40 °C, was added to a deionized water dispersion of VMT (10 g). In the case of ODTMA, the VMT was dispersed in a 50/50 mixture of ethanol/deionized water, and the alkyl ammonium salt was dissolved in ethanol at room temperature. Then, the exchange reactions were carried out at 85 °C and 24 h for ETO and at 60 °C and 24 h for ODTMA. The organoclays were collected by filtration and after being washed repeatedly with hot deionized water. In the case of the ODTMA-modified VMT, the excess surfactant (i.e., unbound surfactant) was then removed by soxhlet extraction with ethanol for one day and then acidified ethanol at pH 4 for 12 h [42].

### 2.3. Preparation of PLA/Vermiculite Nanocomposites

Nanocomposites with 2 and 5 wt % organo-VMTs were prepared by melt compounding with PLA as described elsewhere [40,41]. Dried PLA pellets and organo-VMTs were added in a MiniLab II Haake Rheomix CTW5 co-rotating mini twin-screw extruder (Waltham, MA, USA). The temperature, screw speed, and mixing time were set at 185 °C, 50 rpm, and 5 min. The extruded mixtures were compression-molded at 185 °C and 150 bars of pressure for 5 min in a hot-plate hydraulic press, to obtain plaques. For comparison, unfilled PLA was also processed. Extent of organic modification and code names of the different nanocomposites are listed in Table 1.

### 2.4. Sample Characterization

The nanoscale dispersion of VMTs within PLA was analyzed by transmission electron microscopy (TEM). A Philips Tecnai G2 20 TWIN TEM (Eindhoven, The Netherlands) at 200 kV accelerated voltage was used to obtain the micrographs of the nanocomposites. Film specimens were ultramicrotomed at room temperature to give sections of approximately 100 nm, and the observations were carried out after mounting the slice on a 300 hexagonal mesh copper grid.

The melting and crystallization behaviors of pure PLA and PLA/VMT nanocomposites were investigated with a differential scanning calorimeter, (DSC-Q2000) from TA Instruments (New Castle, DE, USA), calibrated with indium, under ultrapure nitrogen atmosphere. All specimens were weighed to be in the range of 5–6 mg and were first heated from room temperature to 200 °C and held in the molten state for 5 min to erase the previous crystalline thermal history. Then, the samples were cooled to room temperature at 40 °C/min; after 3 min at 25 °C, the samples were reheated to 200 °C at a controlled rate (from 8 to 2 °C/min). Glass transition temperature (*T*_g_), cold crystallization temperature (*T*_cc_), melting temperature (*T*_m_), the cold crystallization enthalpy (∆*H*_cc_), and melting enthalpy (∆*H*_m_) were determined from the second heating scan. 

## 3. Results and Discussion

### 3.1. Characterization of PLA/VMT Nanocomposites

#### Morphology

**Figure 2:**
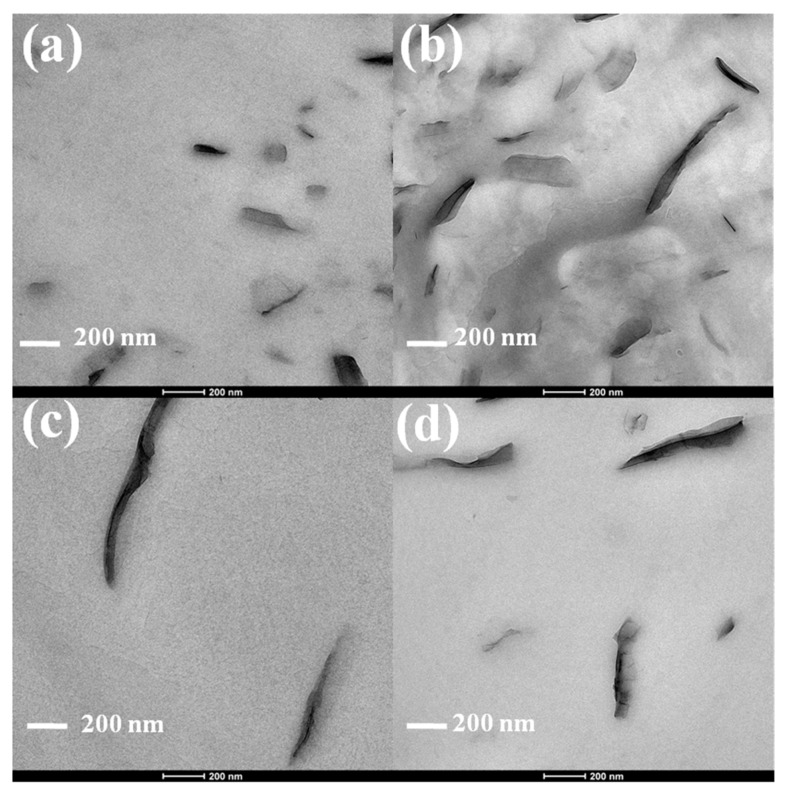
TEM images of (**a**) PLA/ETOVMT-2, (**b**) PLA/ETOVMT-5, (**c**) PLA/C18VMT-2, (**d**) PLA/C18VMT-5.

TEM images of PLA/VMTs are presented in Figure 2. In the micrographs, the dark entities are the cross section of the clay platelets, and the brighter regions represent the polymer matrix. The images of Figure 2a,b reveal the coexistence of disordered intercalated and delaminated clay platelets in the presence of ETOVMT, and it is observed that lower clay loading corresponds to higher extent of exfoliation. For the nanocomposite containing C18VMT, clay layers aggregated in thin stacks and some intercalated stacked clay layers are observed. The formation of a most exfoliated structure is observed in the case of PLA/ETOVMT nanocomposite, indicating that the compatibility with PLA is higher for this organomodified VMT due to favorable interactions of the hydroxyl groups of the intercalated surfactant cations in the clay with carbonyl functions of polymer chains [40,41].

### 3.2. Non-Isothermal Cold Crystallization and Melting Behavior

**Figure 3:**
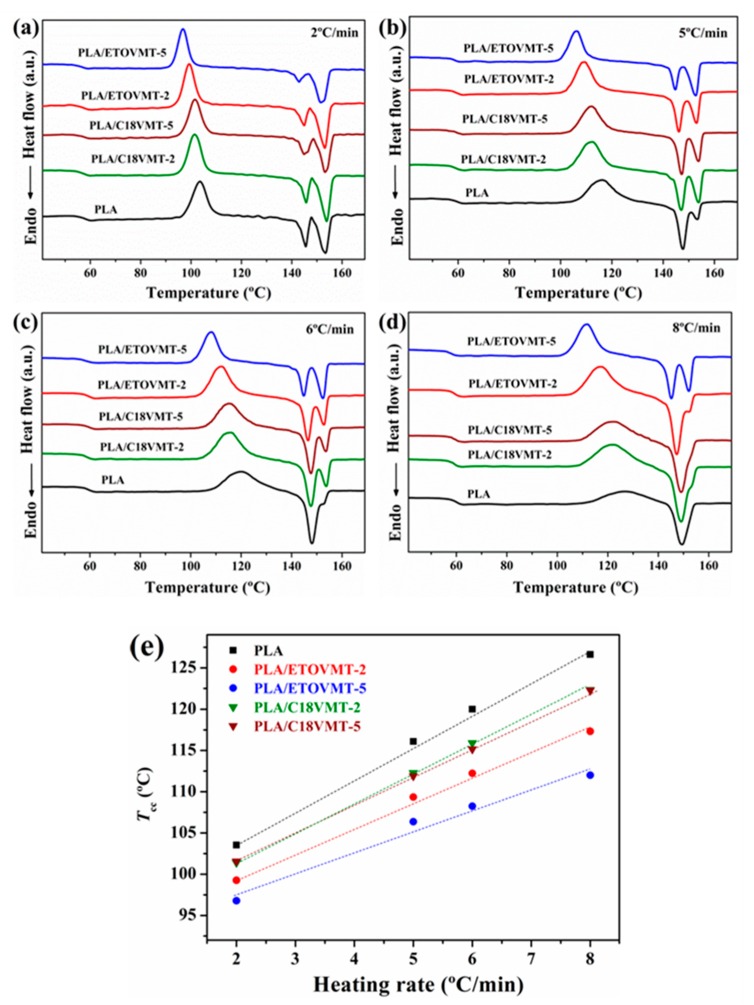
DSC thermograms of neat polylactide (PLA) and PLA/VMT nanocomposites obtained at heating rates (**a**) 2 °C/min, (**b**) 5 °C/min, (**c**) 6 °C/min, and (**d**) 8 °C/min. (**e**) Cold crystallization temperature as a function of the heating rate for PLA and PLA/VMTs.

Figure 3 illustrates the DSC thermograms of neat PLA and PLA/VMT nanocomposites from the second heating scan at various heating rates. In the thermograms, several peaks can be observed for all samples. The first one, the exothermic peak around 60 °C is ascribed to *T*_g_, the second one is the cold crystallization peak, and the last endothermic peak is due to the melting of PLA matrix. The cold crystallization exotherm for neat PLA becomes broader and shifts to higher temperature as the heating rate increases; the same trend is observed for its nanocomposites, especially for those containing 2 wt % VMTs. Two melting peaks appear for PLA and the nanocomposites depending on the heating rate and the type and loading of VMT. The common thermal characteristics, *T*_g_, *T*_cc_, and *T*_m_, of these materials obtained from the thermograms shown in Figure 3 are listed in Table 2. 

*T*_g_ value of PLA is unaffected by the incorporation of VMTs. With increasing heating rate, the temperature when crystallization begins (the onset of crystallization temperature, *T*_co_) and *T*_cc_ shift to higher temperatures for both neat PLA and PLA/VMT composites, meaning that higher heating rates correspond to higher temperatures at which the crystallization process starts. The nucleation and crystal growth are retarded as the heating rate increases. The presence of the two different organomodified VMTs in PLA/VMT nanocomposites leads to a decrease in *T*_co_ and *T*_cc_ at a given heating rate, particularly for the nanocomposite containing ETOVMT (Figure 3, Table 2), indicating that the VMTs make the non-isothermal cold crystallization of PLA easier, since PLA starts to crystallize earlier. This result indicates that nanoclays nucleate PLA cold crystallization.

For the PLA/ETOVMT nanocomposites, *T*_cc_ decreases with the increase of the organomodified VMT content at all heating rates, whereas the *T*_cc_ values of PLA/C18VMT nanocomposites are unaffected by the clay loading. *T*_cc_ values of the PLA/ETOVMT-5 nanocomposite are the lowest, followed by the values of PLA/ETOVMT-2. These results imply that ETOVMT nucleates PLA cold crystallization more effectively than C18VMT, which suggests that the difference in the dispersion state of VMTs represents an important factor in the difference in crystallization behavior of PLA/VMT nanocomposites. Similar results were reported by Fukhusima et al. [35], with the clay exhibiting a better level of dispersion displaying a more efficient nucleation.

∆*H*_cc_ is affected by both the addition of VMTs and heating rate (Table 2 and Figure S1). ∆*H*_cc_ varies between 22.4 and 10 J/g for unfilled PLA, decreasing almost linearly as the heating rate increases. This is due to the fact that at high heating rates, the PLA chains do not have enough time to crystallize, as the temperature increases too fast. For PLA/VMT nanocomposites, the ∆*H*_cc_ value depends on the sample (i.e., the type and content of VMT in the PLA matrix) and the heating rate. The ∆*H*_cc_ value decreases as the heating rate increases from 2 to 5 °C/min and then remains constant with a further increase in the heating rate. The addition of VMTs leads to a decrease in ∆*H*_cc_ at heating rates lower than 8 °C/min and an increase at 8 °C/min, as compared to neat PLA. The ∆*H*_cc_ value of PLA at heating rate of 8 °C/min is around 10 J/g, while that value for the nanocomposites containing organomodified VMTs is between 16–14 J/g. Better degrees of dispersion of the VMT in the PLA corresponded to lower ∆*H*_cc_ values. Moreover, a decrease in ∆*H*_cc_ value is observed with increasing clay loading. At low heating rates, the large number of nuclei formed by the presence of VMT reduces the mobility of PLA chains, restricting crystal growth, and a decrease in the cold crystallization enthalpy is observed. At faster heating rates, the nucleating effect of the VMTs seems to be efficient enough to promote crystallization of PLA. Wu et al. [33], Ublekov et al. [34], and Picard et al. [38] observed similar results from their studies of non-isothermal cold crystallization of PLA/OMMT.

*X*_cc_ was determined using the following equation:

(1) $$X_{\mathrm{cc}}\;=\;\left[\frac{\Delta H_{\mathrm{cc}}}{\Delta H_{m}^{0}\;\times \;w_{\mathrm{PLA}}}\right]\;\times \;100$$

where ∆*H*_cc_ is the crystallization enthalpy of the sample, ∆*H*_m_^0^ is the enthalpy of fusion of a perfect PLA crystal (93 J/g [24]), and *w*_PLA_ is the weight fraction of PLA in the nanocomposite. 

Cold crystallinity values, *X*_cc_, are presented in Table 2 and Figure S2. The *X*_cc_ value of neat PLA decreases almost linearly with the heating rate. However, for PLA/VMT nanocomposites, a decrease in the *X*_cc_ value is observed at 5 °C/min, and it is almost independent of the heating rate in the range of 5 to 8 °C/min. The incorporation of 5 wt % ETOVMT and C18VMT to PLA results in a noticeable decrease in the cold crystallinity value at heating rates lower than 8 °C/min, whereas an increase is observed compared to that of neat PLA at 8 °C/min for all nanocomposites, especially those containing 2 wt % organoclays. Similar variation trends concerning *T*_cc_, ∆*H*_cc_, and *X*_cc_ have been reported in the literature by Wu et al. [33] and Picard et al. [38] in their studies of non-isothermal cold crystallization of PLA/OMMT nanocomposites. Therefore, it can be concluded that VMTs promote the non-isothermal cold crystallization of PLA at high heating rates, and that the degree of dispersion in the polymer matrix and the clay content are significant factors in the different crystallization behavior of PLA.

The melting behavior of PLA and its nanocomposites containing VMTs depends on the heating rate and the clay type and loading (Figure 3). The endothermic peak of unfilled PLA splits into two peaks (*T*_m1_ and *T*_m2_) during heating at low heating rates (2 and 5 °C/min), whereas when heating at 6 and 8 °C/min a single endotherm is observed at a temperature intermediate between those of the double melting peaks. As regards nanocomposites, the presence of one or two melting peaks depends on the type and content of vermiculite and the heating rate. The nanocomposite containing 5 wt % ETOVMT exhibits two melting peaks at all heating rates, whereas those with a content of 2 wt % ETOVMT or C18VMT and 5 wt % C18VMT display two melting peaks at heating rates less than 8 °C/min. The melting–recrystallization–melting processes of PLA lamellae lead to a double endothermic melting peak [23]. The fusion of thin lamellae formed during the cold crystallization process results in the first endothermic peak, whereas the fusion of lamellae formed through the melting–recrystallization of primary thin lamellae gives rise to the second melting peak. The lamellae structure of PLA in the nanocomposites is reflected by the first endothermic peak. Picard et al. [38] also observed the presence of two melting peaks for a larger range of heating rates in the case of the PLA/OMMT nanocomposites in comparison with neat PLA. 

**Figure 4:**
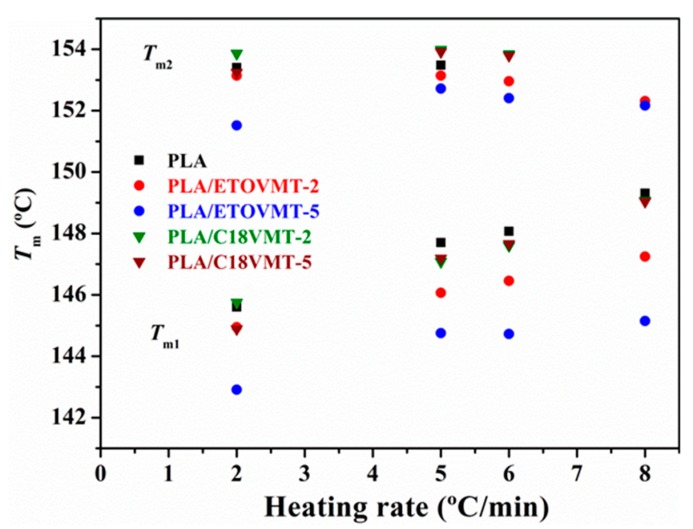
Melting temperatures of PLA and PLA/VMT nanocomposites as a function of the heating rate.

The variation of melting temperatures for pure PLA and PLA/VMT nancomposites with the heating rate is presented in Figure 4. The temperature of the first endothermic peak (*T*_m1_) increases as heating rate increases, whereas the value of the second peak (*T*_m2_) either remains constant or decreases slightly. PLA/ETOVMT nanocomposites exhibit the lowest *T*_m1_ values at all heating rates, and this temperature decreases with increasing the organoclay content. On the other hand, the *T*_m1_ values of the PLA nanocomposites containing C18VMT are close to that of neat PLA. These results confirm that ETOVMT acts as nucleating agent, promoting heterogeneous nucleation resulting in less perfect crystallites compared to those of pure PLA, whereas the incorporation of C18VMT has no effect on the crystallite perfection. The presence of VMTs and the dispersion degree have an effect on cold crystallization and melting behavior of PLA matrix. Nam et al. [32], Wu et al. [33], Fukhusima et al. [35], and Picard et al. [38] reported much more defective crystals in the PLA/clay nanocomposites than in the case of pure PLA.

*X*_m_ was determined using the following equation:

(2) $$X_{m}\;=\;\left[\frac{\Delta H_{m}}{\Delta H_{m}^{0}\;\times \;w_{\mathrm{PLA}}}\right]\;\times \;100$$

The variation of *X*_m_ with heating rate for PLA and its nanocomposites is shown in Figure S3. *X*_m_ of PLA and PLA/C18VMT nanocomposites decreases almost linearly as heating rate increases, whereas for PLA/ETOVMT nanocomposites *X*_m_ decreases significantly when the heating rate increases from 2 to 5 °C/min, and it remains practically constant during further heating rate increase. The addition of organomodified VMTs to PLA results in an increase in the crystallinity value at 8 °C/min as compared to that of neat PLA. 

**Figure 5:**
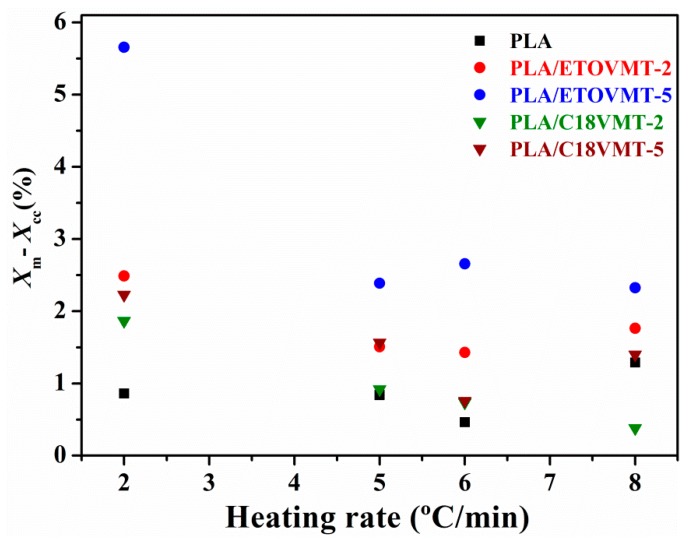
Crystallinity of PLA and PLA/VMT nanocomposites as a function of the heating rate.

The actual crystallinity of PLA and its nanocomposites, calculated as the difference between *X*_m_ and *X*_c_, as a function of heating rate is presented in Figure 5. The *X*_m_–*X*_c_ values of PLA decrease as the heating rate increases, at low heating rates the molten PLA has more time to reorganize itself into new crystals. The presence of organomodified VMTs has only a small effect on *X*_m_–*X*_c_ values of PLA. The incorporation of 5 wt % of ETOVMT leads to a slightly higher value of *X*_m_–*X*_c_ compared with that of neat PLA at all heating rates, most notably at a low heating rate (2 °C/min). DSC analysis data reported by Ublekov et al. [34] showed that the crystallinity of PLLA/Cloisite 30B composites increased drastically at high clay loadings (5–9 wt %).

### 3.3. Non-Isothermal Crystallization Kinetics

In order to understand the effect of the addition of different VMTs on the bulk crystallization rate of PLA, the non-isothermal crystallization kinetics for neat PLA and PLA/VMT nanocomposites were also studied.

For non-isothermal crystallization, the relative degree of crystallinity, *X*_T_, as a function of temperature can be expressed as:

(3) $$X_{T}\;\;=\;\;\frac{\int_{T_{0}}^{T}(dH_{c}/dT)dT}{\int_{T_{0}}^{T_{\infty }}(dH_{c}/dT)dT}$$

where *T*_0_ and *T*_∞_ represent the onset and the end of crystallization temperature, respectively; *T* is any temperature in the crystallization process, and *dH*_c_ represents the differential crystallization enthalpy change in temperature range of *dT*.

From DSC crystallization curves of neat PLA and PLA/VMT composites, the evolution of the relative crystallinity as a function of temperature at all different heating rates are shown in Figure S4. All data gave sigmoidal-type curves showing that higher heating rates correspond to higher temperatures needed to initiate the crystallization. 

**Figure 6:**
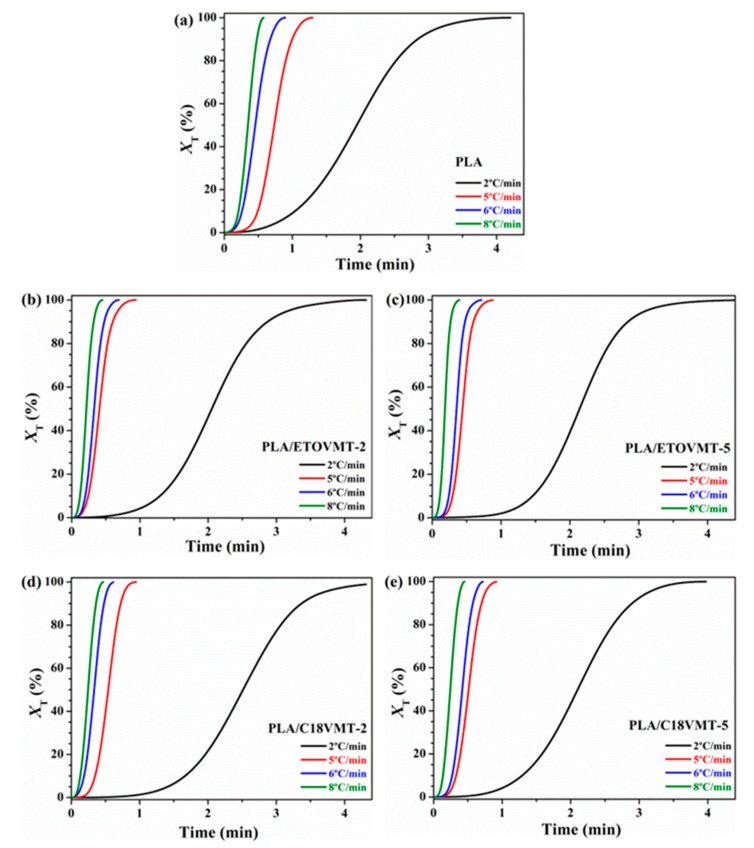
Relative crystallinity as a function of time at different heating rates for the cold crystallization of (**a**) PLA, (**b**) PLA/ETOVMT-2, (**c**) PLA/ETOVMT-5, (**d**) PLA/C18VMT-2, (**e**) PLA/C18VMT-5.

Once the *X*_T_ is obtained, conversion into *X*_t_ can be carried out by transforming the temperature axis to time axis using the transformation:

(4) $$t\;\;=\;\;\frac{T_{0\;}\;-\;T}{\phi }$$

where *T* is the temperature at the crystallization time *t*, and *ϕ* is the heating rate. The relative crystallinity data thus obtained (*X*_t_) are illustrated in Figure 6. 

The higher the heating rate is, the shorter the time required for crystallization process will be. In these S-type crystallization curves an induction period can be observed initially, corresponding to the primary nucleation process, which is followed by a rapid increase in crystallization in which crystal growth occurs, which can be noticed from the linear ascending part, and ultimately a decrease in crystallization rate, but the crystallinity continues to increase slowly. This last action is recognized by the part that deviates from the curve and refers to secondary crystallization.

**Figure 7:**
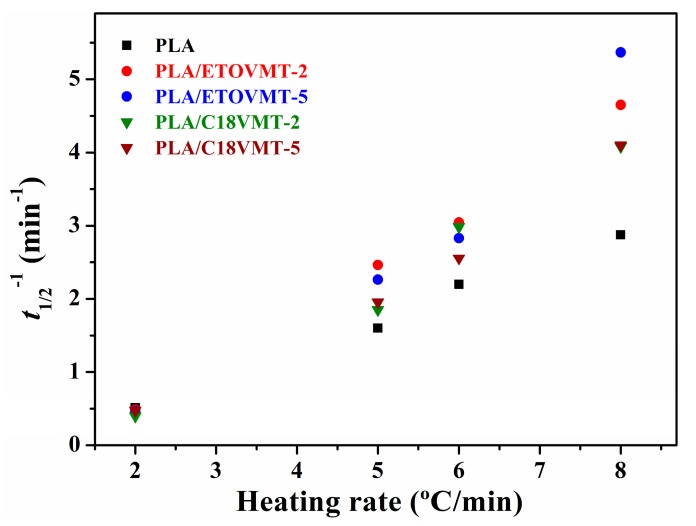
Reciprocal half-time of crystallization at different cooling rates for PLA and PLA/VMT nanocomposites.

The crystallization half-time (*t*_1/2_), the time required to achieve 50% of the final crystallinity of the samples, can be calculated directly from the plots of relative crystallinity versus crystallization time (Figure 6). Such value is very important to discuss the non-isothermal cold crystallization rate of neat PLA and its nanocomposites. The inverse value of *t*_1/2_ (i.e., 1/*t*_1/2_) signifies the bulk crystallization rate, and a lower 1/*t*_1/2_ value indicates slower crystallization. The 1/*t*_1/2_ value depends on the cooling rate, and as expected it decreases with increasing heating rate for both PLA and PLA/organo-VMT nanocomposites (Figure 7), indicating that higher heating rate corresponds to faster crystallization process. The 1/*t*_1/2_ values in the nanocomposites are higher than that of neat PLA at the heating rates higher than 2 °C/min, indicating that the nanoclay could accelerate the overall crystallization process of PLA. However, at 2 °C/min heating rate the values of 1/*t*_1/2_ for PLA/organo-VMT composites are similar to that of neat PLA, suggesting that at this heating rate the addition of organoclay does not affect the rate of crystallization. The maximum difference between the 1/*t*_1/2_ value of pure PLA and PLA/VMT nanocomposites is observed at the highest heating rate (i.e., 8 °C/min). The PLA/ETOVMT nanocomposites exhibit the highest value of the reciprocal of *t*_1/2_, indicating that the crystallization processes of PLA/ETOVMT nanocomposites are finished in shorter times than that of pure PLA, since the rate of the crystallization of PLA is faster in the presence of ETOVMT due to its nucleating effect.

**Figure 8:**
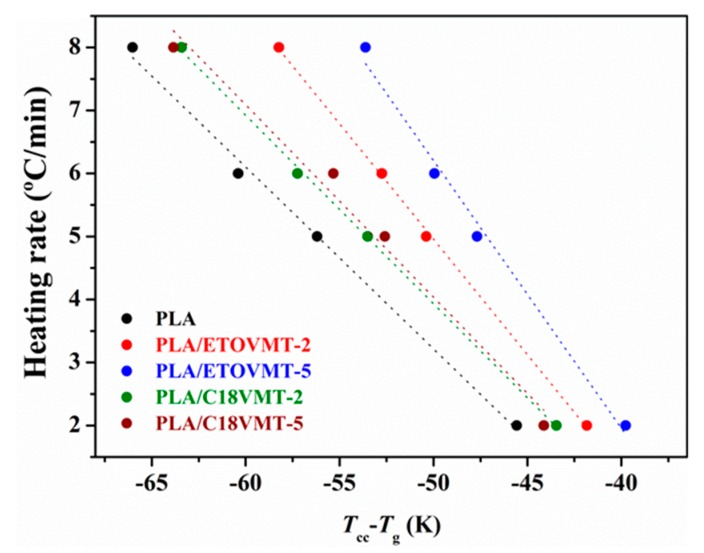
Plots of heating rate as a function of the difference between the cold crystallization and glass transition temperatures for PLA and PLA/VMT nanocomposites.

**Figure 9:**
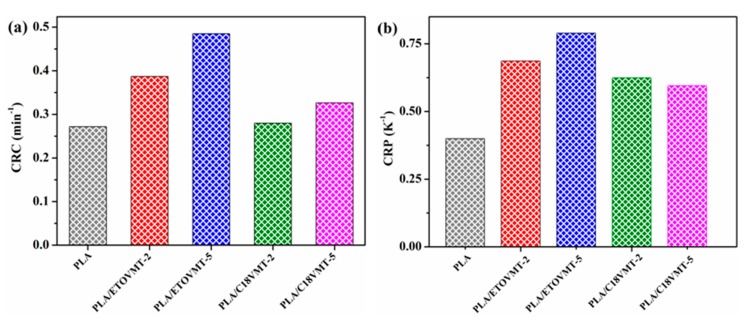
(**a**) Crystallization rate coefficient (CRC) and (**b**) crystallization rate parameter (CRP) values of PLA and PLA/VMT nanocomposites.

In order to evaluate the effect of VMTs on the crystallization rate of PLA in the nanocomposites quantitatively, two approaches can be used: (1) the “crystallization rate coefficient” (CRC) and (2) the “crystallization rate parameter” (CRP). The CRC, defined as the variation in cooling rate required to change the undercooling of the polymer melt of 1 °C, was suggested by Khanna [43]. The CRC values should be higher for faster crystallizing systems. The CRC can be determined from the slope of a line by plotting the cooling rate against *T*_m_ − *T*_p_, where *T*_m_ and *T*_p_ are the melting point and non-isothermal melt crystallization peak temperature, respectively. In the present work, crystallization behaviors of neat PLA and PLA/VMT nanocomposites were studied from the amorphous state; therefore, the determination of CRC has been carried out using *T*_g_ − *T*_p_ instead of *T*_m_ − *T*_p_, representing a change in heating rate to require to bring about 1 °C change in the superheating of the polymer amorphous phase (Figure 8). The CRP, proposed by Zhang et al. [44], can be determined from the slope of the plot of 1/*t*_1/2_ versus cooling rate (Figure 7). Faster crystallization rate corresponds with higher slope. The values of CRP and CRC are displayed in Figure 9.

The CRC value for neat PLA is 0.272 min^−1^; for the nanocomposites this value depends on the VMT type and content, increasing as VMT content increases. The incorporation of 2 wt % C18VMT does not affect the CRC value of PLA, whereas it increases slightly by the addition of 5 wt % C18VMT. However, the enhancement in CRC value is more pronounced in the presence of 2 and 5 wt % ETOVMT. It is inferred that the better the degree of clay dispersion in the PLA matrix is, the higher the CRC value will be. From these results, it can be concluded that the cold crystallization rate of PLA increases when adding 2 and 5 wt % ETOVMT and 5 wt % C18VMT, due to the nucleating effect of the organoclays. The CRP value for PLA (0.3995 K^−1^) is lower than that in the presence of 2 and 5 wt % ETOVMT (0.6868 and 0.7895 K^−1^), and PLA nanocomposites containing 2 and 5 wt % C18VMT (0.5420 and 0.5955 K^−1^) suggesting that the nanocomposites are more crystallizable than PLA. The PLA nanocomposites containing ETOVMT exhibit higher CRC and CRP values than those containing C18VMT, and PLA/ETOVMT-5 shows the highest values, which means that this content of this organomodified VMT is the most effective in accelerating the cold crystallization of PLA, and that the higher the degree of dispersion is, the faster the crystallization process will be. Fukhusima et al. [35] reported similar results, showing that the better the level of dispersion of the clay was, the faster the rate of crystallization would be.

The common approach used to analyze the isothermal crystallization kinetics is the Avrami equation, which allows the calculation of the crystallinity fraction, *X*_t_, as a function of the crystallization time *t* [45,46]:

(5) $$1-\;X_{t}\;=\;\;\exp\;(-Z_{t}\;t^{n}\;)$$

where *X*_t_ is the crystallinity fraction in the crystallizable material at time *t*. *Z*_t_ and *n* are constants typical of a given morphology and type of nucleation. *Z*_t_ is the crystallization rate constant and is temperature dependent; *n* is the Avrami index and contains information on nucleation and growth geometry.

To study kinetic parameters for non-isothermal crystallization processes, several methods have been developed and the majority of the proposed formulations are based on the Avrami equation. One approach is the use of Avrami analysis for data obtained under non-isothermal measurements. Equation (5) is converted to the following equation:

(6) $$\ln\;\left\{-\ln\;\left[1-X_{t}\right]\right\}\;=\;\;n\;\;\ln\;t\;\;+\;\;\ln\;Z_{t}\;$$

Ozawa [47] extended the Avrami Equation to the non-isothermal condition, taking into account the effect of the cooling rate, and derived a Kinetic Equation as follows:

(7) $$1-X_{t}=exp\left[\frac{K\left(T\right)}{\phi^{m}}\right]$$

where *X*_t_ is the relative crystallinity, *K*(*T*) represents the cooling function that depends on the temperature of the process, which is related to overall crystallization rate and indicates the speed at which crystallization occurs, ϕ is the cooling rate and *m* is the Ozawa exponent depending on the crystal growth and nucleation mechanism.

**Figure 10:**
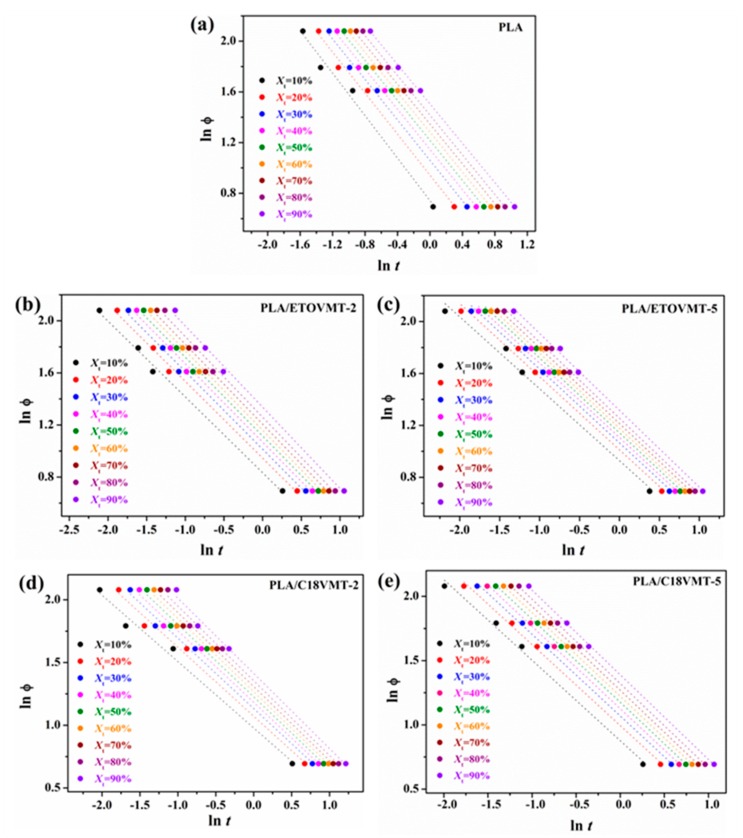
Plots of ln ϕ vs. ln *t* for the non-isothermal cold crystallization of (**a**) PLA, (**b**) PLA/ETOVMT-2, (**c**) PLA/ETOVMT-5, (**d**) PLA/C18VMT-2, (**e**) PLA/C18VMT-5.

Mo and coworkers [48] proposed an approach to study the non-isothermal crystallization of polymer systems by combining the Avrami Equation (5) with the Ozawa Equation (7). For the non-isothermal crystallization process, physical variables relating to the process are *X*_t_, heating rate, and crystallization temperature. By rearranging the Avrami Equation at a given crystallinity *X*_t_:

(8) ln ϕ =  ln  F(T)  − α  ln t 

where the parameter *F*(*T*) = [*K*(*T*)/*K*]^1/m^ refers to the value of the heating rate chosen at a unit crystallization time, when the system has a certain degree of crystallinity. The *F*(*T*) value has a definite physical and practical meaning; that is, at a certain value of *X*_t_, a high value of *F*(*T*) is needed to reach this *X*_t_ value in a unit of time. *F*(*T*) reflects the difficulty of the crystallization process. The smaller the value of *F*(*T*) is, the higher the crystallization rate will be. Here, α is the ratio of the Avrami exponent *n* to Ozawa exponent *m* (α = *n*/*m*). According to Equation (8), at a given degree of crystallinity the plot of ln ϕ against ln *t* should yield a straight line with an intercept of ln *F*(*T*) and a slope of—α. Figure 10 shows the plots of neat PLA and its nanocomposites at different relative crystallinity values. The linearity of the plots of Figure 10 indicates that the non-isothermal cold crystallization kinetics of PLA and PLA/VMT nanocomposites can be fitted by Mo’s model. The values of *F*(*T*), α and correlation coefficients r^2^ are listed in Table 3. 

The values of *F*(*T*) increase with increasing the relative crystallinity for PLA and PLA/VMT nanocomposites, indicating that at unit crystallization time, to obtain a higher degree of crystallinity a higher heating rate should be used. In other words, the polymer chain movement slows down, hindering the formation of crystals. The values of α are almost constant, close to 0.8 for neat PLA, and to 0.60 for the nanocomposites. The values of *F*(*T*) for all PLA/VMT nanocomposites are smaller than those for neat PLA at relative crystallinity higher than 20%, suggesting that crystallization rate of nanocomposites is higher than that of PLA at conversions higher than 20%. Wu et al. [33] also reported a higher crystallization rate for intercalated PLA/OMMT nanocomposites than neat PLA from the kinetics results obtained by applying the Mo model.

### 3.4. Crystallization Activation Energy

Effective activation energy is an important parameter associated with non-isothermal crystallization, as it determines the rate of the process. The effective activation energy (∆*E*) for cold crystallization under continuous heating conditions can be estimated by the Kissinger Equation [49]:

(9) $$\frac{d\;\;\left[\ln\;\left(\frac{\phi }{T_{p}^{2}}\right)\right]\;}{d\;\left(\frac{1}{T_{p}}\right)}\;\;=\;\;-\frac{\Delta E}{R}\;$$

where ϕ, *E*, *R*, and *T*_p_ are the heating rate, activation energy, gas constant, and the crystallization peak temperature, respectively. Plots of ln(ϕ/*T*_p_^2^) versus 1/*T*_p_ for PLA and PLA/VMT nanocomposites yield straight lines with a slope of ∆*E*/*R* (Figure S5a). The results of the Kissinger plots for neat PLA and its nanocomposites are listed in Table 4. Besides the Kissinger equation, the Takhor model [50] is another widely used non-isothermal method. According to the Takhor model, ∆*E* can be determined with the following equation:

(10) $$\frac{d\;\;\left[\ln\left(\phi \;\right)\right]\;}{d\;\left(\frac{1}{T_{p}}\right)}\;\;=\;\;-\frac{\Delta E}{R}\;$$

The slope of the lines from plots of Figure S5b determines ∆*E*/*R*. The results obtained from the Takhor model are listed in Table 4. 

∆*E* is the activation energy required to transport molecular segments to the crystallization surface, and the values are negative, indicating that the rate of crystallization increases as the temperature decreases. The ∆*E* value for PLA crystallization was in the range of 70–76 kJ/mol, which is similar to that reported by Wu et al. and obtained by the Kissinger equation [33]. The two models show that the ∆*E* value of neat PLA is lower than those of PLA/VMT nanocomposites, suggesting that clays make the motion of the PLA chain segments more difficult. Moreover, ∆*E* for nanocomposites increases with increasing clay loading, and the highest values are attained in the presence of ETOVMT, indicating that the incorporation of ETOVMT to PLA causes more restriction in the transportation of polymer chains during crystallization due to the more uniform dispersion of clay platelets compared to C18VMT. Similar results were obtained by Pluta [16] from the study of the effect of dispersion degree of the silicate layers on the cold crystallization behavior of PLA/Closite 30B by DSC. Wu et al. [33] also reported a monotonous small increase in ∆*E* of PLA/OMMT with increasing clay content.

The crystallization process of polymer nanocomposites is affected by two main factors: nucleation and the restriction of molecular mobility of polymer chains. The crystallization rate is determined by the competition of these two factors. However, taking into account the reduction in the crystallization half-time, the increase in CRC and CRP values, and the lower values of kinetic parameter (*F*(*T*)) from Mo’s model, it appears that the nucleation dominates over the retarded mobility of PLA chains, leading to an accelerated crystallization process.

## 4. Conclusions

The study has explored the effect of different organic modifiers on the non-isothermal cold crystallization behavior and kinetics and the melting behavior of PLA/organomodified vermiculites by DSC. A different dispersion state of the clay particles in PLA was obtained depending on the organic modifier used. The best clay platelets dispersion was obtained in the presence of ETO as organic modifier. The results revealed that the cold crystallization of PLA was affected by the clay loading and the extent of compatibility between organovermiculite and the PLA matrix. The nucleation effect of vermiculite resulted in the decrease of *T*_cc_ of PLA at all heating rates. The most compatible organovermiculite (ETOVMT) was found to exhibit the most pronounced nucleating effect. The cold-crystallinity of PLA decreased at heating rates ≤5 °C/min particularly upon incorporation of high clay loadings, while an increase was observed at 8 °C/min, especially at low clay contents. As a result of the fusion–recrystallization–melting processes of the PLA lamellae, double melting peaks appeared, in a wider range of heating rates for the nanocomposites compared to PLA. ETOVMT led to the less perfect crystallites. The Mo model satisfactorily described the cold crystallization kinetics of pristine PLA and PLA/vermiculite nanocomposites. Crystallization half-time, non-isothermal crystallization kinetic parameters derived from the Mo model, and the CRC and CRP approaches demonstrated that the PLA/organovermiculite nanocomposites crystallized more easily than neat PLA due to the nucleating effect of clays. The results showed that the nanocomposite with the highest loading of the most compatible organovermiculite crystallized with a faster crystallization rate. The activation energy for non-isothermal cold crystallization of PLA, evaluated by the Kissinger and Takhor methods, was lower than that of PLA/organovermiculite nanocomposites. The nucleation effect of the clays dominated over the restrictions of polymer chain mobility.

## Figures and Tables

**Table 1:** Characteristics of clays and nanocomposites, designation, and clay content.

Clay Code	Organic Modifier	Organic Content (%)	Sample Code	wt % of VMT
				In Nanocomposites
ETOVMT	ETO	28.1	PLA/ETOVMT-2	2
			PLA/ETOVMT-5	5
C18VMT	ODTMA	4.5	PLA/C18VMT-2	2
			PLA/C18VMT-5	5

**Table 2:** DSC results derived from the second heating scan for pure PLA and PLA/VMTs.

Sample	ϕ (°C·min^−1^)	*T*_g_ (°C)	*T*_co_ (°C)	*T*_cc_ (°C)	∆*H*_cc_ (J·g^−1^)	*X*_cc_ (%)	*T*_m1_ (°C)	*T*_m2_ (°C)	∆*H*_m_ (J·g^−1^)	*X*_m_ (%)
PLA	2	57.9	97.3	103.5	22.4	24.1	145.6	153.4	23.2	24.9
	5	59.9	107.0	116.1	17.4	18.7	147.7	153.5	18.2	19.6
	6	59.6	108.0	120.0	16.6	17.9	148.1		17.1	18.4
	8	60.6	110.2	126.6	10.1	10.8	149.3		11.3	12.1
PLA/ETOVMT-2	2	57.4	94.7	99.2	22.9	24.5	144.9	153.1	24.3	26.6
	5	58.9	102.8	109.3	16.3	17.2	146.1	153.1	17.0	18.7
	6	59.5	104.7	112.2	16.2	17.1	146.5	152.9	16.9	18.5
	8	59.1	107.6	117.3	16.1	16.9	147.2	152.3	17.1	18.7
PLA/ETOVMT-5	2	57.0	92.7	96. 8	18.6	19.0	142.9	151.5	21.8	24.7
	5	58.7	100.6	106.4	13.5	13.8	144.7	152.7	14.3	16.2
	6	58.3	101.9	108.2	13.9	14.2	144.7	152.4	14.9	16.9
	8	58.4	104.3	112.0	13.7	13.9	145.1	152.2	14.4	16.3
PLA/C18VMT-2	2	57.9	96.3	101.1	24. 8	26.1	145.7	153.9	26.5	27.9
	5	58.8	104.0	112.3	16.4	17.3	147.1	153.9	17.3	18.2
	6	58.7	107.0	115.9	15.5	16.3	147.6	153.8	16.2	17.1
	8	58.9	110.1	122.3	15.1	15.9	149.1		15.5	16.3
PLA C18VMT-5	2	57.4	96.6	101.6	20.7	21.1	144. 9	153.2	22.8	23.3
	5	59.3	104.3	111.9	15.1	15.5	147.2	153.9	16.7	17.0
	6	59.8	107.0	115.2	14.9	15.2	147.7	153.8	15.6	16.0
	8	58.4	110.1	122.3	15.1	12.5	149.0		13.6	13.91

*X*_cc_: crystallinity of cold crystallization; *T*_m1_, *T*_m2_: the low and high-temperature melting endotherms; *X*_m_: crystallinity related to the heat of fusion.

**Table 3:** The non-isothermal crystallization kinetic parameters based on the Mo model for PLA and PLA/VMT nanocomposites.

Sample	*X*_t_ (%)	10	20	30	40	50	60	70	80	90
PLA	α	0.84	0.81	0.80	0.79	0.79	0.79	0.78	0.78	0.78
	*F*(*T*)	2.10	2.57	2.89	3.15	3.38	3.61	3.84	4.12	4.51
	r^2^	0.9889	0.9935	0.9953	0.9964	0.9969	0.9976	0.9983	0.9989	0.9995
PLA/ETOVMT-2	α	0.58	0.59	0. 60	0.60	0.61	0.61	0.61	0.62	0.62
	*F*(*T*)	2.30	2.57	2.76	2.91	3.05	3.19	3.34	3.53	3.81
	r^2^	0.9969	0.9968	0.9965	0.9965	0.9963	0.9962	0.9964	0.9963	0.9967
PLA/ETOVMT-5	α	0.55	0.59	0.60	0.57	0.58	0.58	0.61	0.62	0.59
	*F*(*T*)	2.53	2.57	2.76	3.02	3.14	3.25	3.34	3.53	3.73
	r^2^	0.9906	0.9968	0.9965	0.9959	0.9967	0.9971	0.9964	0.9963	0.9982
PLA/C18VMT-2	α	0.58	0.59	0.60	0.57	0.58	0.58	0.61	0.62	0.60
	*F*(*T*)	2.69	2.57	2.75	3.26	3.41	3.55	3.34	3.53	4.11
	r^2^	1	0.9968	0.9965	0.9934	0.9937	0.9935	0.9964	0.9963	0.9933
PLA/C18VMT-5	α	0.58	0.65	0.59	0.64	0.65	0.65	0.61	0.62	0.66
	*F*(*T*)	2.69	2.70	2.76	3.08	3.24	3.40	3.34	3.53	4.02
	r^2^	1	1	0.9965	0.9996	0.9998	0.9999	0.9964	0.9963	0.9997

**Table 4:** The crystallization activation energy calculated from Kissinger and Takhor models for PLA and PLA/VMTs.

	∆*E* (kJ·mol^−1^)	
Sample	Kissinger	Takhor
PLA	−69.7	−76.3
PLA/ETOVMT-2	−87.8	−94.2
PLA/ETOVMT-5	−102.5	−108.8
PLA/C18VMT-2	−76.5	−82.9
PLA/C18VMT-5	−110.1	−83.9

## Supplementary Materials

The following are available online at https://www.mdpi.com/2073-4360/12/2/364/s1, Figure S1: Cold crystallization enthalpy as a function of the heating rate for PLA and PLA/VMTs; Figure S2: Cold crystallinity of PLA and PLA/VMTs nanocomposites as a function of the heating rate; Figure S3: Melting crystallinity of PLA and PLA/VMTs nanocomposites as a function of the heating rate; Figure S4. Relative crystallinity as a function of temperature at different heating rates for the cold crystallization of (a) PLA, (b) PLA/ETOVMT-2, (c) PLA/ETOVMT-5, (d) PLA/C18VMT-2, (e) PLA/C18VMT-5; Figure S5. (a) Kissinger and (b) Takhor plots for the non-isothermal cold-crystallization of PLA and PLA/VMTs nanocomposites.
