# Supplementary material for: Effect of Organic Modifier and Clay Content on Non-Isothermal Cold Crystallization and Melting Behavior of Polylactide/Organovermiculite Nanocomposites
Source: Polymers (Basel). 2020 Feb 7;12(2):364. doi: 10.3390/polym12020364 (PMC7077447; doi:10.3390/polym12020364)
Supplement: Supplementary file 1 [file polymers-12-00364-s001.pdf]

polymers

MDPI

Supplementary Materials

Effect of Organic Modifier and Clay Content on Non-Isothermal Cold Crystallization and Melting Behavior of Polylactide/OrganoVermiculite Nanocomposites

M. Jesús Fernández and M. Dolores Fernández*

Department of Polymer Science and Technology. Faculty of Chemistry, University of the Basque Country UPV/EHU, Paseo Manuel Lardizábal 3, 20018 San Sebastián, Spain; mjesus.fernandez@ehu.es (M.J.F.)
* Correspondence: mariadolores.fernandez@ehu.es

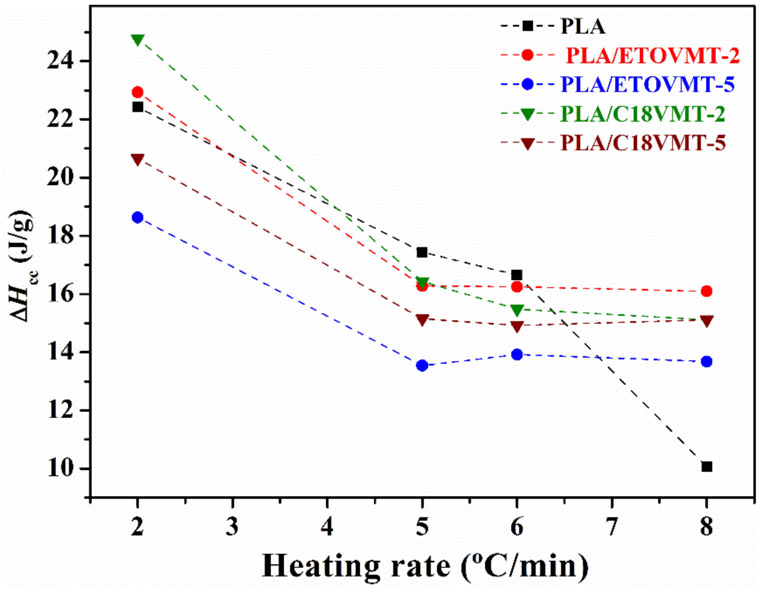

Figure S1. Cold crystallization enthalpy as a function of the heating rate for PLA and PLA/VMTs.

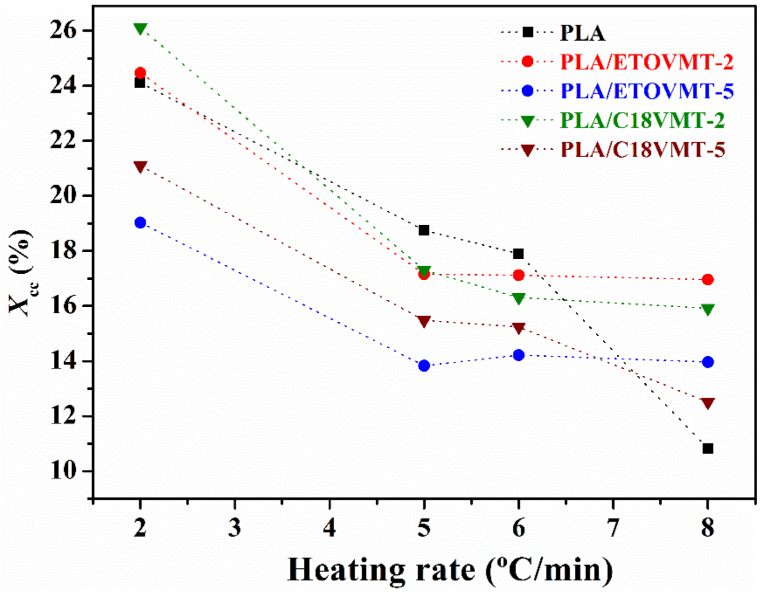

Figure S2. Cold crystallinity of PLA and PLA/VMTs nanocomposites as a function of the heating rate.

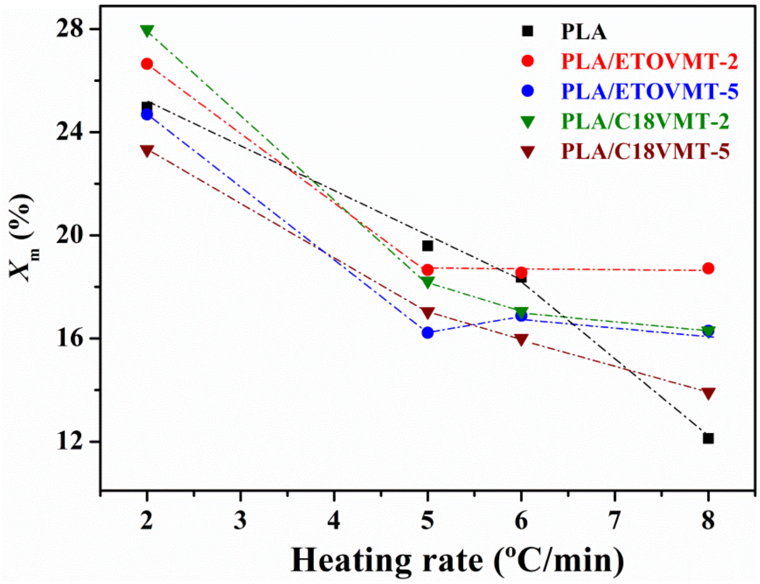

Figure S3. Melting crystallinity of PLA and PLA/VMTs nanocomposites as a function of the heating rate.

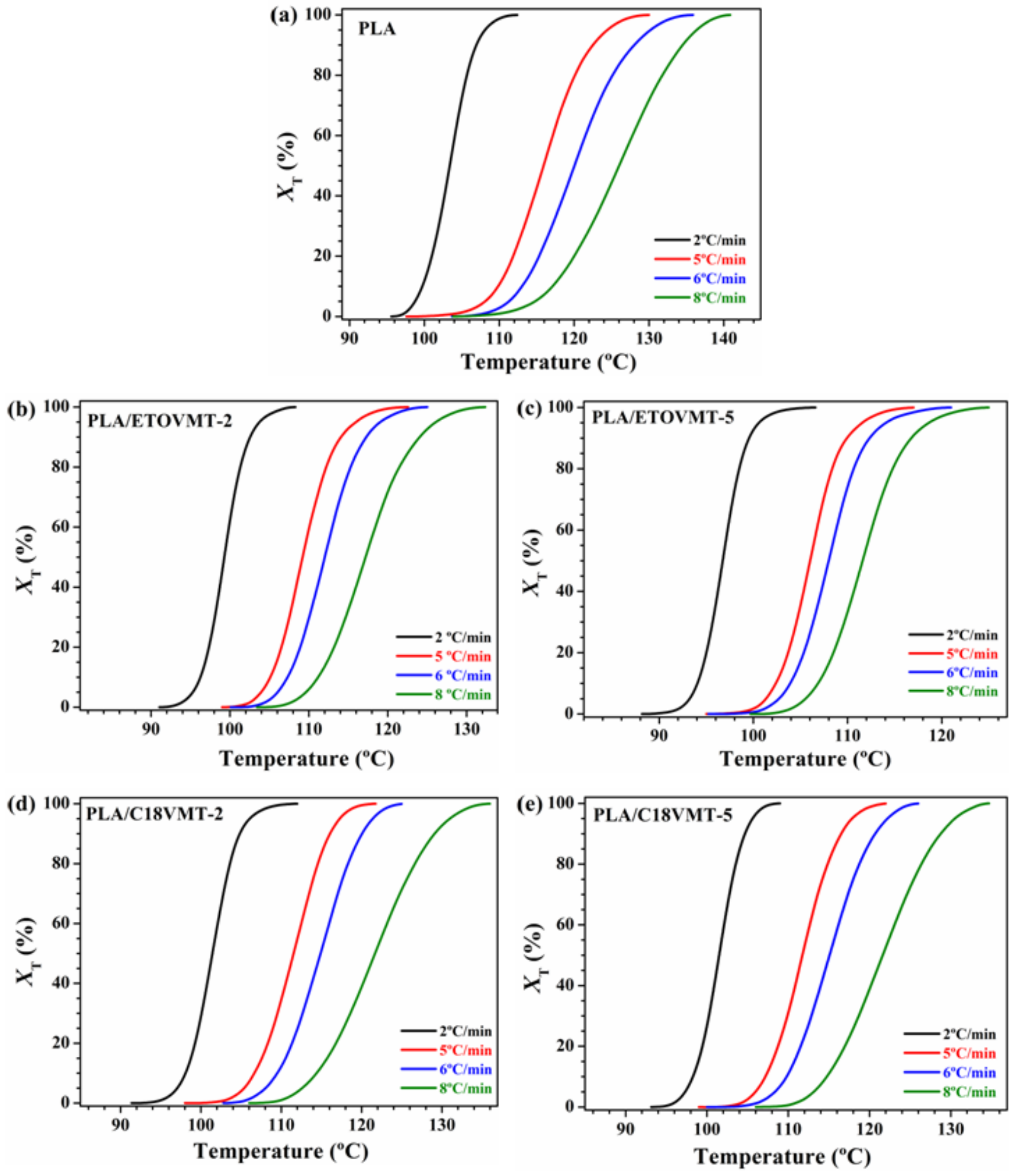

Figure S4. Relative crystallinity as a function of temperature at different heating rates for the cold crystallization of (**a**) PLA, (**b**) PLA/ETOVMT-2, (**c**) PLA/ETOVMT-5, (**d**) PLA/C18VMT-2, (**e**) PLA/C18VMT-5.

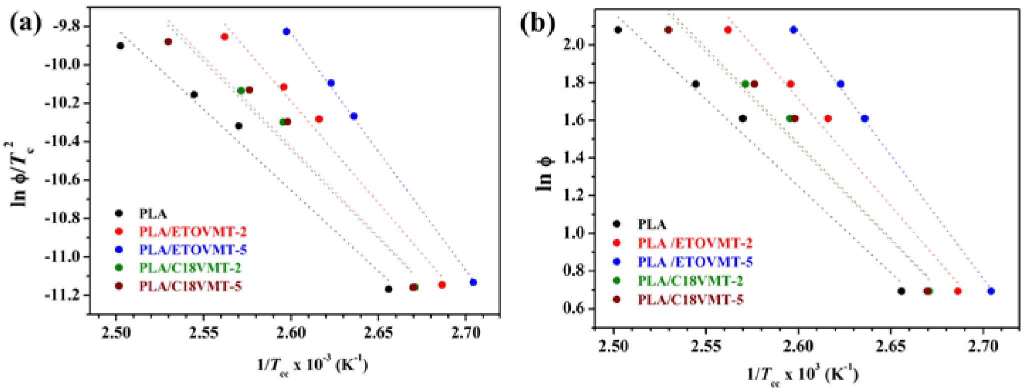

Figure S5. (**a**) Kissinger, (**b**) Takhor plots for the non-isothermal cold-crystallization of PLA and PLA/VMTs nanocomposites.
